# Instantaneous mutation rate in cancer initiation and progression

**DOI:** 10.1186/s12918-018-0629-z

**Published:** 2018-11-22

**Authors:** Shuhao Sun, Fima Klebaner, Xinan Zhang, Tianhai Tian

**Affiliations:** 1 School of Mathematical Sciences, Monash University, Melbourne, 3800 VIC Australia; 2 School of Mathematics and Statistics, Central China Normal University, Wuhan, 430079 People’s Republic of China

**Keywords:** Cancer, Mathematical model, Initial mutation rate, Cancer cell doubling time

## Abstract

**Background:**

Cancer is one of the leading causes for the morbidity and mortality worldwide. Although substantial studies have been conducted theoretically and experimentally in recent years, it is still a challenge to explore the mechanisms of cancer initiation and progression. The investigation for these problems is very important for the diagnosis of cancer diseases and development of treatment schemes.

**Results:**

To accurately describe the process of cancer initiation, we propose a new concept of gene initial mutation rate based on our recently designed mathematical model using the non-constant mutation rate. Unlike the widely-used average gene mutation rate that depends on the number of mutations, the gene initial mutation rate can be used to describe the initiation process of a single patient. In addition, we propose the instantaneous tumour doubling time that is a continuous function of time based on the non-constant mutation rate. Our proposed concepts are supported by the clinic data of seven patients with advanced pancreatic cancer. The regression results suggest that, compared with the average mutation rate, the estimated initial mutation rate has a larger value of correlation coefficient with the patient survival time. We also provide the estimated tumour size of these seven patients over time.

**Conclusions:**

The proposed concepts can be used to describe the cancer initiation and progression for different patients more accurately. Since a quantitative understanding of cancer progression is important for clinical treatment, our proposed model and calculated results may provide insights into the development of treatment schemes and also have other clinic implications.

## Background

Pancreatic cancer is one of the most aggressive malignancies in humans, with a five-year relative survival rate of only 8% patients [1, 2]. This disease occurs when the damaged cells grow in an uncontrolled manner. The most common treatment options for pancreatic cancer patients include surgery, endoscopic treatment, chemotherapy and radiation therapy [3]. The design of a treatment plan is based on a number of factors, including the severity and spread of the tumour, as well as the patient’s health conditions and age [4, 5]. Due to the location of the pancreas which is deep within the abdomen, this type of cancer is difficult to diagnose and is often found at an advanced stage. The observed aggressive malignancy of this disease may be due to the factors such as the delay in diagnosis or early metastatic dissemination [6–8]. Therefore, it is important to conduct experimental and theoretical studies for the initiation of this disease and the dynamics of cancer progression, which may be helpful to diagnose this disease at an earlier stage [9].

It is widely recognised that cancer diseases are initiated from gene mutations that increase the fitness of cancer cells over that of the surrounding normal cells [10–12]. The recent advances in high-throughout technologies and systems biology approaches have provided huge amount of data showing the mutation heterogeneity in cancer cells [13–16]. A number of theoretical studies have been proposed to investigate the initiation and progression of cancer diseases [17–19]. Mathematical modelling has been used as a powerful tool to elucidate mechanisms in cancer initiation and progression [20–23]. These models have provided quantitative predictions that may be validated by experimental or clinical studies. For example, regarding the mechanisms of metastacis, a modelling study has suggested that more than 10 years might be needed to generate the first parental, non-metastatic founder cell from the first gene mutation [18]. In addition, more than five years may be needed for cancer cells to achieve the metastatic ability.

A key assumption in the mathematical models for cancer study is that the gene mutation rate is constant, which is defined as the number of mutations over a fixed time period. The value of mutation rate is obtained using the ratio of mutation number to the number of cells in the population [17, 18]. However, experimental studies suggests that the mutation rate of a cell may be dependent on the number of gene mutations inside the cell, though it is still in the debate regarding the effects of driver mutation and passenger mutation in gene mutation and cell growth [24, 25]. Growing evidence suggests that gene mutations can be deleterious to cancer cells and play an important role in both cancer progression and clinical treatment. In the process of tumorigenesis and the metastasis of pancreatic cancer, at least 3–7 driver mutations have been identified in the clinical and experimental studies. Experimental studies suggest that the telomeric shortening and mutations in Kras gene are among the earliest and most pervasive alterations [26–28].

In a previous study, we have proposed a model using the non-constant gene mutation rate [29]. However, this initial study did not provide the detailed method for determining the variations of gene mutations in different patients. In this work, based on the non-constant mutation rate, we further propose the concept of the gene initial mutation rate and instantaneous tumour double time. Using the data of seven patients, we calculate the initial mutation rate of each patient. Then we calculate the tumour size using the instantaneous tumour double time.

## Implementation and results

### Mathematical model

In this work we consider a dynamic model to study the process of cancer initiation and progression. Based on the proposed assumptions in [29], we use a continuous function of time to represent the gene mutation rate, whose derivative is a linear function of the number of mutations occurred, namely

1 $$ \mu\triangleq\frac{dN}{dt}=a+bN,   $$

where *N* is the number of mutations, *μ*(*t*) is the mutation rate at time *t*, *a* is the mutation rate of normal cells (namely the cells without any gene mutation) and *b* is a constant. The solution of Eq. (1) with regarding to *N* is

$$N(t)=ce^{bt}-\frac{a}{b}, $$

where *c* is an arbitrary constant. Note that this solution is valid if *b*≠0. Then the mutation rate can be written as

$$\mu(t)=bce^{bt} $$

when *b*≠0. Combined with the case of *b*=0 (namely *μ*=*a*), the mutation rate is denoted as

2 $$ \mu(t)=\mu(0)e^{bt}.  $$

Here mutation rate *μ*(0) is termed as the initial mutation rate which is the mutation rate for cells without any gene mutation. If the value of *b* is zero, the mutation rate is a constant which has been widely used in the literature.

**Fig. 1:**
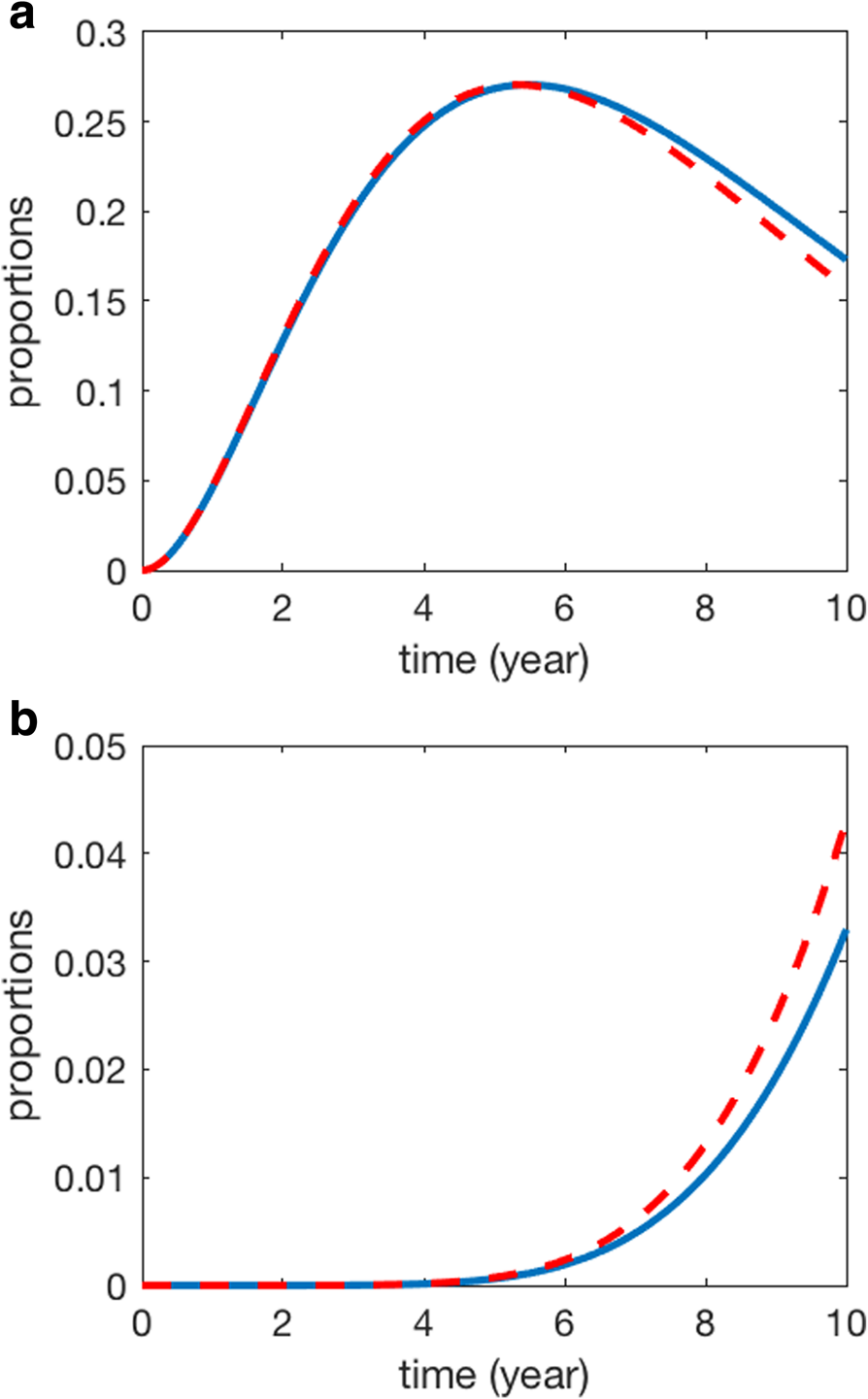
Numerical simulations of model (4) using the constant mutation rate and non-constant mutation rate. **a** proportions of cells with two mutations. **b** proportions of cells with eight mutations. (Solid line: model using constant mutation rate; dash-red line: model using non-constant mutation rate)

We now consider a model for the dynamics of cell population with different numbers of gene mutations. Let *p*_*j*_(*t*) be the fraction of cancer cells with *j* mutations at time *t*. In addition, *t*_*j*_ is the time point when the first cancer cell with exact *j* mutations appears. Here we assume that *t*_0_=0. When using the continuous mutation rate *μ*(*t*), the system is modelled by

3 $$\begin{array}{@{}rcl@{}} \frac{dp_{0}(t)}{dt}&=&-\mu(t)p_{0}(t),\\ \frac{dp_{j}(t)}{dt}&=&-\mu(t)p_{j}(t)+\mu(t)p_{j-1}(t),\\ \frac{dp_{k}(t)}{dt}&=&\mu(t)p_{k-1}(t), \end{array} $$

where *j*=1,...,*k*−1. Figure 1 provides simulations of model (3) based on either a constant mutation rate (namely *μ*(*t*)=*const*) or a rate function of time *t* (2). Here we considered a cancer system with a maximal number of 8 mutations. Figure 1 suggest that the difference between simulations obtained by the two types of mutation rates is small if the number of mutations *k* is small (see Fig. 1a). However, Fig. 1b shows that the difference may be large when the mutation number is large. In this simulation, *a*=0.000001, *b*=0.00003, and the initial condition is *p*_0_(0)=1 and *p*_*i*_(0)=0 (*i*=1,…,8).

Now we consider another realization for the non-constant gene mutation rate. It is assumed that the rate is a piecewise linear function, namely the value of mutation rate is approximated by a constant

$$\mu(t)\approx \mu\left(t_{j}\right)\equiv\mu_{j} $$

during the interval [*t*_*j*−1_,*t*_*j*_] between the two consecutive mutations. Using this piece-wise mutation rate, we have the following model [29]

4 $$\begin{array}{@{}rcl@{}} \frac{dp_{0}(t)}{dt}&=&-\mu_{0}p_{0}(t),\\ \frac{dp_{j}(t)}{dt}&=&-\mu_{j}p_{j}(t)+\mu_{j-1}p_{j-1}(t),\\ \frac{dp_{k}(t)}{dt}&=&\mu_{k-1}p_{k-1}(t), \end{array} $$

where *j*=1,...,*k*−1.

The solution of system (3) is given by [29]

$$p_{j}(t)=\frac{\lambda(t)^{j}e^{-\lambda(t)}}{j!}, $$

where

$$\lambda(t)=\int_{0}^{t}\mu(x)dx=\frac{\mu_{0}}{b}\left(e^{bt}-1\right). $$

Then the time point *t*_*j*_, at which the first *j*-mutated cell occurs, satisfies

5 $$ t_{j}=\frac{ln\left(\frac{b\lambda_{j}}{\mu_{0}}+1\right)}{b}   $$

where *λ*_*j*_=*λ*(*t*_*j*_).

The clinic data may provide the gene mutation number *N* and the time point when the *N*-th mutation occurs only. That is why the average mutation rate has been widely used in studies. However, it is not obvious to determine the initial mutation rate and parameter *b* in the non-constant mutation rate model based on this limited information. The major contribution of this work is to derive the relationship between the initial mutation rate *μ*(0), parameter *b*, mutation number *N* and average mutation rate.

### Determination of non-constant gene mutation rate

Now we derive a formula to calculate the value of exponent *b* and gene initial mutation rate *μ*(0) based on the gene mutation number *N* and average mutation rate $\overline {\mu }$. We first consider the average mutation rate, defined by

$$\overline{\mu}_{N}=\frac{N}{t_{N}}. $$

By using Eq. (5), the average mutation rate is given by

$$\overline{\mu}_{N}=\frac{bN}{ln\left(b\lambda_{N}/\mu_{0}+1\right)}. $$

Thus the value of *μ*_0_ is given by

6 $$ \mu_{0}=\frac{b\lambda_{N}}{e^{bN/\overline{\mu}_{N}}-1}  $$

Then comsidering the following equations:

$$\mu_{j}=\mu\left(t_{j}\right)=\mu_{0}e^{bt_{j}}=b\lambda_{j}+\mu_{0}, $$

where *j*=1,...,*N*, the analytical solution for *b* may not exist. Thus our goal is to find an approximation of *b* with good accuracy.

To this purpose, we consider the sequence $\{u_{j}=\mu _{0}e^{t_{j}b}, t=1,2,...,N\}\phantom {\dot {i}\!}$ which is a geometric series. The mean of this series has the form

$$\begin{array}{@{}rcl@{}} \overline{\mu}^{\prime}_{N} &=&\frac{1}{N}\sum\limits_{j=1}^{N} u_{j}\\ &=& \frac{\mu_{0}\left(1-e^{bt_{N}}\right)e^{b}}{\left(1-e^{b}\right)t_{N}} \end{array} $$

and it satisfies

$$\overline{\mu}^{\prime}_{j}\leq \overline{\mu}_{j}, $$

for *j*=1,...,*N*. Substituting the expression (5) of *t*_*N*_ into the above equation, we have that

$$\overline{\mu}^{\prime}_{N} =\frac{-b^{2}\lambda_{N} e^{b}}{\left(1-e^{b}\right)ln\left(\frac{b\lambda_{N}}{\mu_{0}}+1\right)}  $$

and hence

7 $$ \mu_{0}=\frac{b\lambda_{N}}{\frac{b^{2}\lambda_{N} e^{b}}{e^{\left(e^{b}-1\right)\overline{\mu}_{N}^{\prime}}}-1}.   $$

Again from $t_{N}={N}/{\overline {\mu }_{N}}$ and

$$\ln\left(\frac{b\lambda_{N}}{\mu_{0}}+1\right)=\frac{bN}{\overline{\mu}_{N}} $$

by using (5), we have that

$$\frac{bN}{\overline{\mu}_{N}} = \frac{-b^{2}\lambda_{N} e^{b}}{\left(1-e^{b}\right)\overline{\mu}^{\prime}_{N}} $$

and

$$\frac{N}{\overline{\mu}_{N}} = \frac{\lambda_{N} e^{b} b}{\overline{\mu}^{\prime}_{N}\left(e^{b}-1\right)}. $$

Note that when *b* is very small, we have that

$$\frac{-b e^{b}}{1-e^{b}} \approx 1. $$

To find the value of *b* with good accuracy, we assume that

8 $$ \frac{\overline{\mu}^{\prime}_{j}}{\overline{\mu}_{j}} = \frac{\lambda_{j}}{j}=e^{-bN/\overline{\mu}_{N}},   $$

where *j*=1,2,…,*N*−1, and *λ*_*N*_=*N*. Thus from Eq. (7), we again obtain Eq. (6), which implies that assumption (8) is reasonable. On the other hand, using the notation

$$\overline{\lambda}_{N}=\frac{1}{N}\sum\limits_{j=1}^{N}\lambda_{j}, $$

then we have

$$\overline{\lambda}_{N} = \frac{N+1}{2}e^{-\frac{bN}{\overline{\mu}_{N}}}. $$

Finally, we derive the following theorem by using the assumption (8).

#### **Theorem 1**

The relationship between the average mutation rate $\overline {\mu }$, number of mutations *N* and parameter *b* is given by

9 $$ \overline{\mu}_{N}=e^{-bN/\overline{\mu}_{N}} \frac{b(N+1)}{2}+\frac{bN}{e^{bN/\overline{\mu}_{N}}-1}.   $$

#### *Proof*

The average mutation rate satisfies □

10 $$ \overline{\mu}_{N} = b\overline{\lambda}+\mu_{0}=\frac{b(N+1)}{2e^{Nb/\overline{\mu}_{N}}}+\mu_{0}.   $$

Thus this theorem is proved by using Eqs. (6) and (10).

Note that, when the values of $\overline {\mu }_{N}$ and *N* are given, we can find the value of *b* by solving the nonlinear Eq. (9). We use MAPLE to solve this equation and obtain the value of *b*.

### Initial mutation rate

To demonstrate the importance of non-constant mutation rate, we first calculate various mutation rates based on the clinic data of seven pancreatic cancer patents [18]. Table 1 gives the information regarding the survival time from diagnosis, age at diagnosis and gene mutation numbers of these seven patients. The calculated values of parameter *b* for the seven patients are also given in Table 1. In addition, we calculate the initial mutation rate using the total number of mutations, parameter *b* and average mutation rate, given by

11 $$ \mu(0)=\overline{\mu}-\frac{e^{-bN/(2\overline{\mu})} b(N+1)}{2}  $$

**Table 1:** Estimated average mutation rate, initial mutation rate and the value of parameter *b* based on the clinic data of seven patients from [18] (Survival from diagnosis: month)

Patients	Survival from diagnosis	Clone time diagnosis	Mutations	Mutation rate	Initial rate	b
Pa01C	6	9.8	49	0.0192	0.01917	0.00001
Pa02C	8	9.4	35	0.019	0.0189	0.000018
Pa03C	1	2.4	28	0.0223	0.0201	0.000030
Pa04C	7	7.9	34	0.0188	0.0185	0.000019
Pa05C	10	4.3	28	0.0194	0.0189	0.000029
Pa07C	3	3.1	50	0.0198	0.0198	0.00001
Pa08C	15	10.6	35	0.0193	0.0190	0.000018

**Fig. 2:**
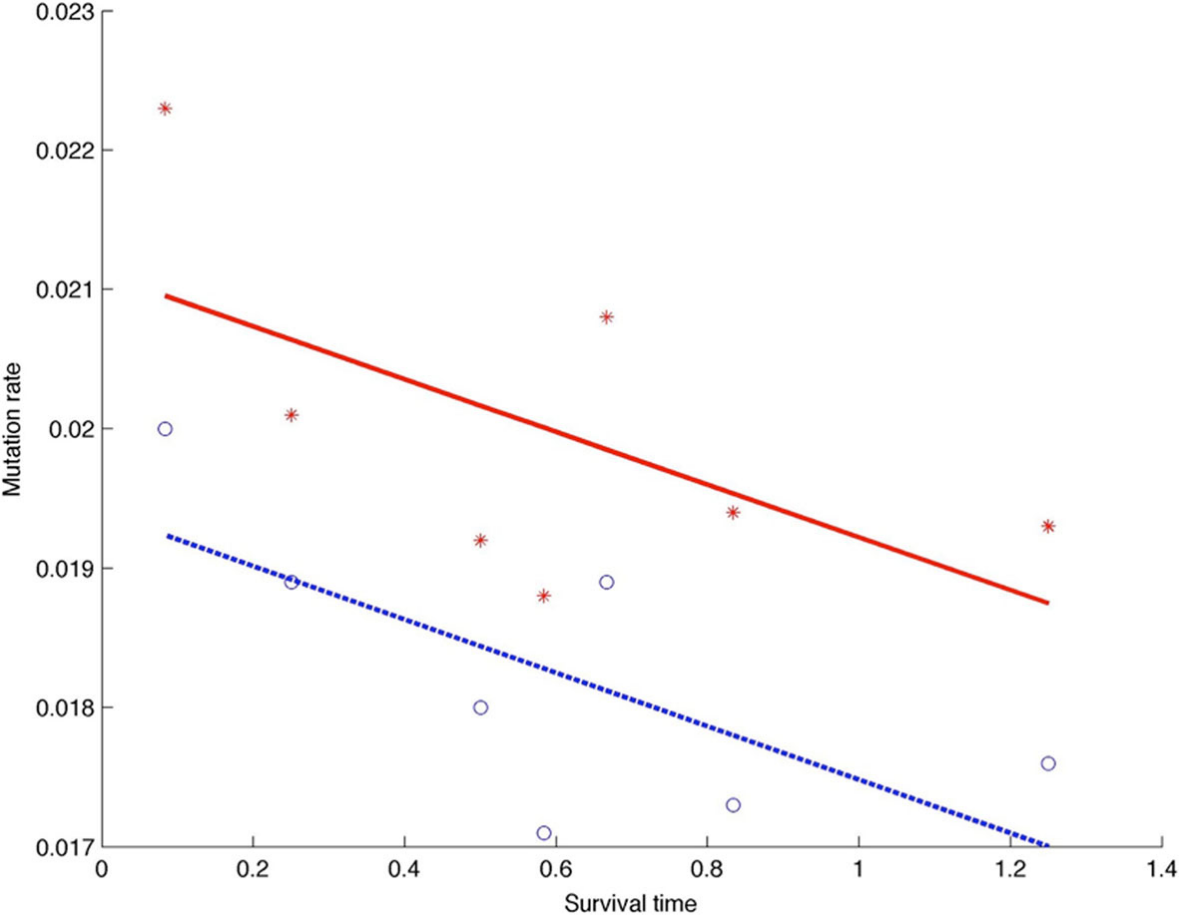
Negative correlations between the patient suvivour time and gene mutation rate (Circle: initial mutation rate, blue under line: predicted initial mutation rate; star: average mutation rate, red above line: predicted average gene mutation rate)

Table 1 suggests that the average mutation rate and initial mutation rate both are negatively correlated with the survival time of patients from prognosis. This means that the smaller the mutation rate is, the longer the patient survives. For example, the three smaller initial mutation rates are 0.0172, 0.173 and 0.0176 for patients Pa04C, Pa05X and Pa08C, respectively. The corresponding survival time periods of these three patients are the longest ones, namely 6, 7 and 15 months, respectively. On the other hand, the highest initial mutation rate 0.020 (patient Pa03C) corresponds to the shortest survival time (namely one month). The initial mutation rate can be expressed as a function of the survival time of patients, given by

12 $$ \mu_{0}=a[\mathrm{S}]+b,  $$

where [*S*] is the survival time of patients from diagnosis. We use the least square regression method to find the values of coefficients of *a* and *b* which are −0.0019 and 0.0194, respectively. The values in Fig. 2 clearly show the negative correlation between the initial mutation rate and survival time of patients.

Note that the averaged mutation rate is also negatively correlated with the survival time. The next question is whether the average mutation rate can give a better regression relationship with the survival time. To answer this question, we use a similar function

13 $$ \overline{\mu} = c{\text{[S]}}+d  $$

to represent the relationship; and calculate the values of *c* and *d* which are −0.0019 and 0.0211, respectively. Although Fig. 2 suggests that the average mutation rate also has a consistent negative correlation with the survival time, the scaled mean-square error, defined by

$$\text{Error} = \frac{1}{\text{mean}(\mu)}\sqrt{\sum\limits_{i=1}^{n}\left(\mu_{i}-\mu_{i}^{*}\right)^{2}},$$

where *μ*_*i*_ is the estimated mutation rate in Table 1 for patient *i* and $\mu _{i}^{*}$ is the predicted mutation rate by using either the regression (12) or (13). The error for the predicted initial mutation rate and that for average mutation rate is 0.1007 and 0.1197, respectively. This result suggests that the initial mutation rate provides a better indicator for patient survivor time than the average mutation rate. We have also find the correlation regression relationship between the survival time and the age of patient, as well as the relationship between the survival time and gene mutation number. Numerical results suggest that these variables are not as good as the gene mutation rate for the indicator of survival time (Results not shown).

### Instantaneous tumour double time

Pancreatic cancer has an extremely poor prognosis. Factors that appear to be important in predicting long-term survival following resection include clear surgical margins, small tumour size (2 cm), negative lymph nodes, and reduced perioperative morbidity [7, 30, 31]. It is well known that metastasis accounts for 90% of cancer deaths [32]. The challenge is whether we can detect the tumour during the stage T1 (namely the time between tumour initiation and the birth of the cell giving rise to the parental clone), or even after stage T1 but before seeding of metastases. Advanced imaging methods, as well as other test methods to detect cancer-specific proteins, transcripts, or genes, tumor markers, offer hopes for such non-invasive early detection.

To address this issue, we study the time required for cancer cells successfully to leave the primary tumour based on our non-constant mutation rate. We first refine the notion of tumour doubling time (DT) and propose the concept of instantaneous tumour double time, which is defined as the average tumour double time over a very short period of time. Although the concept of tumour doubling time is widely used for the quantification of tumour growth rate, it involves a number of factors for the growth of cancer cells, such as the tumour type, growth stage, presence of symptoms, and the patient’s lifestyle. Thus it is not easy to determine and analyse the instantaneous tumour doubling time accurately. Based on the research results in [5], Yachida et al. considered the doubling time curve as a piecewise linear function [18]. It assumes that the cell division time is 2.3 days for pancreatic cancer when the tumour size is less than 1 mm, and after that (namely the tumour size is larger than 1 mm) the cell division time is 56 days [18]. The key factor behind this assumption is angiogenesis. Here we consider a continuous curve which fits the Amikura-Yachida curve with good accuracy.

Since it is widely recognized that the cell growth rate is proportional to the number of driver mutations inside the cell, the doubling time should be proportional to the nonlinear gene mutation rate. Based on the Amikura-Yachida model, we propose the following smooth curve which describes the number of doubling as a function of time *t*,

14 $$ \text{DT}=\frac{\mu_{0}}{\overline{\mu}_{0}}\exp\left(\left(b-\overline{b}\right)t\right)\frac{at}{K+t},  $$

where *μ*_0_ and *b* are the initial mutation number and mutation parameter of a particular patient, $\overline {\mu }_{0}$ and $\overline {b}$ are the average parameters based on the corresponding values of all patients, and *a* and *K* are parameters to match the data in [18]. Thus it is clear the proposed method can make more accurate prediction if the number of patients is larger.

To estimate the values of *a* and *K*, we remove the initial mutation rate and parameter *b*, the average value of the doubling time is given by

15 $$ \text{DB}=\frac{at}{K+t}.  $$

**Fig. 3:**
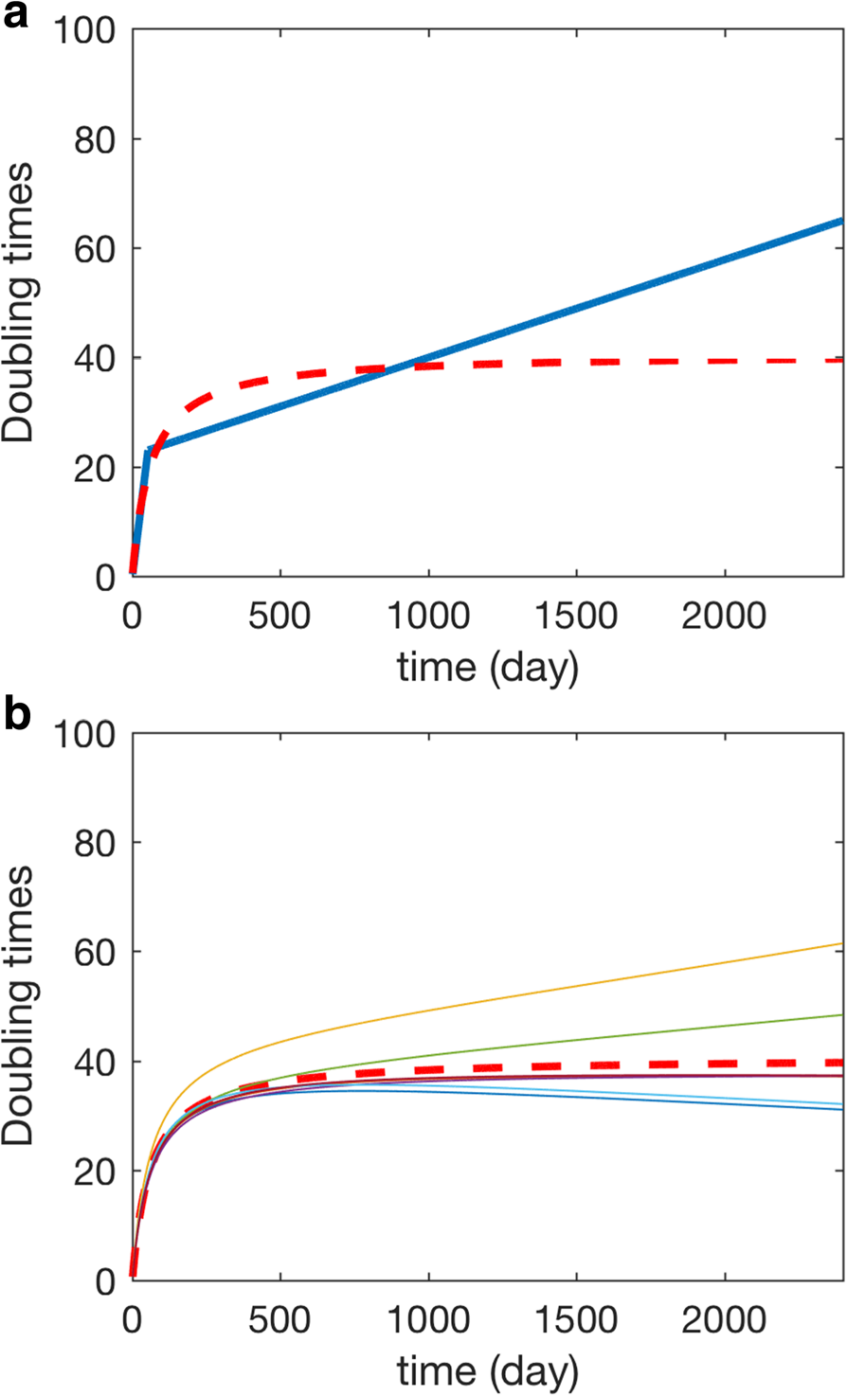
The number of cell doubling over time. **a** The Amikura-Yachita curve using piece-wise doubling time (solid-line) and our proposed average nonlinear model (15) (dot-line). **b** The doubling time curve for seven patients using the proposed nonlinear model (14)

We use this function to realize 23 doubling time in 53 days and the following 23 doubling time in the following 1288 days, which is defined by the Amikura-Yachida model. Using the least-square regression method, the estimated values are *a*=40.74 and *K*=62.04. Figure 3a gives the Amikura-Yachida curve and our approximated curve (14). When *t*<53 days, the difference between these two curves is small. However, when *t*>53 days, this approximated curve provides a more reasonable prediction regarding the doubling time. Using the estimated values of *a* and *K* together with the gene mutation rate of each patient, Fig. 3b gives the estimated doubling time of the seven patients.

### Tomour growth curves in metastasis

We have established a formula to calculate the tumour doubling time for each patient. Next we use it to predict the tumour size. It was proposed that the development of tumour can be divided into three stages, namely stage 1 for the development of the first cancer cell, stage 2 for the development of the first cancer cell that has the ability to escape from the original tumour position, and stage 3 for the full development of metastasis. For the seven patients in [18], Yachida et al. estimated the time intervals between stage 1 and stage 2, which are presented in Table 2. For these given data, we calculate the low, medium and high tumour sizes based on the low, average and high values of the time, respectively. These values are the estimated tumour sizes of each patient at the beginning of stage 2. For example, for patient Pa01C, the low, average and high values of the time are 4.05, 4.995 and 5.94 years, respectively, and the estimated tumour size are 4.55, 4.73 and 4.82 cm, respectively. Table 2 also gives the scaled time for the tumour size to reach 2cm based on the estimated average tumour size (M). For example, for patient Pa01C, the time to reach tumour size 2cm is 0.22×4.995=1.01 years. For all patients, the time for reaching 2cm is less than 1/3 of the clone time.

**Table 2:** Estimated tumour size for seven patients. Tumour-size (L) (M) (H) is based on the lower bound, average time and higher bound of the clone time

Patients	Pa01c	Pa02c	Pa03c	Pa04c	Pa05x	Pa07c	Pa08c
Clone time(y)	4.05-5.94	3.3-4.84	3.9-5.72	3.45-5.06	3.3-4.8	3.7-5.4	3.1-4.6
Tumor-size(L)	4.55	4.12	9.08	3.52	4.63	5.95	4.20
Tumor-size(M)	4.73	4.46	10.15	3.80	5.15	6.25	4.58
Tumor-size(H)	4.82	4.71	11.09	4.01	5.59	6.42	4.86
Time for 2cm	0.22	0.28	0.17	0.325	0.275	0.19	0.29

Time for 2cm is the scaled time (based on the average time) for tumour to teach 2cm

Numerical results in Table 2 show that the lowest value of tumour size for these seven patients all are greater than 2cm. Our calculation presents a theoretic support to the empirical tumour size (<2 cm) hypothesis in [5]. In addition, Japan Pancreatic Cancer Registry [4] reported 11,317 patients with carcinoma of the pancreas during the past decade and 3743 patients underwent pancreatectomy. The 5-yr survival rate of all patients undergoing pancreatectomy, including those with malignant islet cell tumour and cystadenocarcinoma, was 16.6%. The 5-yr survival rate of patients with carcinoma of the pancreas of 2 cm or less, excluding malignant islet cell tumour and cystadenocarcinoma, was 36.2% after pancreatectomy. This registry shows that survival rate is on the increase in patients with carcinoma of the pancreas after pancreatectomy, especially in resettable cases with tumour of 2 cm or less. Further improvement in survival rate is expected in carcinoma of pancreas. Our quantitative analysis provides a theoretic support to the above conclusion.

## Discussion and conclusion

In this work, we have proposed new concepts for the initial mutation rate and instantaneous tumour doubling time based on our recently designed mathematical model using non-constant mutation rates. These concepts are aimed at replacing the widely used definitions of the average mutation rate and average doubling time. The proposed initial mutation rate is independent of the mutation number which determines the average mutation rate. Our regression results have suggested that, compared with the average mutation rate, the estimated initial mutation rate has a larger value of correlation coefficient with the patient survival time. In addition, our instantaneous tumour doubling time is a continuous function of time and considers the effect of initial mutation rate and non-constant mutation rate. Thus, compared with the existing model in which the doubling time is a piece-wise linear function, our continuous model may be able to provide more reasonable estimate of tumour growth process. Since a quantitative understanding of cancer progression is important for clinical treatment, our proposed model and calculated results may provide insights into the dynamics of cancer metastasis and hence have clinic implications.

Since the gene mutation rate is usually very small, gene mutations are normally observed in experiments over a long time period. The estimated values of the mutation rate constant in literature all are based on the number of gene mutations over the given time span. Thus, the average mutation rate strongly relies on the observed number of gene mutations. The calculated mutation rate is highly stochastic due to the very small value of mutation number. Our proposed continuous model for the mutation rate considered not only the total mutation number but also the average dynamics of gene mutation which is realized by the model of cancer cell progression. The major contribution of this work is to derive an analytic expression for the instantaneous mutation rate based on the total number of mutation.

## Availability and requirements

**Project name**: Not applicable.

**Project home page**: Not applicable.

**Operating system(s)**: Not applicable.

**Programming language**: MATLAB.

**Other requirements**: Not applicable.

**License**: Not applicable.

**Any restrictions to use by non-academics**: Not applicable.
